# Development of a scale for capturing psychological aspects of physical–digital integration: relationships with psychosocial functioning and facial emotion recognition

**DOI:** 10.1007/s00146-023-01646-9

**Published:** 2023-03-22

**Authors:** Daiana Colledani, Pasquale Anselmi, Egidio Robusto

**Affiliations:** Department of Philosophy, Sociology, Education and Applied Psychology, University of Padova, Via Venezia 14, 35131 Padua, Italy

**Keywords:** Physical–digital integration, Bifactor model, Facial emotion recognition, Anxiety, Depression, Satisfaction with social relationships

## Abstract

The present work aims at developing a scale for the assessment of a construct that we called “physical–digital integration”, which refers to the tendency of some individuals not to perceive a clear differentiation between feelings and perceptions that pertain to the physical or digital environment. The construct is articulated in four facets: identity, social relationships, time–space perception, and sensory perception. Data from a sample of 369 participants were collected to evaluate factor structure (unidimensional model, bifactor model, correlated four-factor model), internal consistency (Cronbach’s *α*, McDonald’s *ω*), and correlations of the physical–digital integration scale with other measures. Results showed that the scale is valid and internally consistent, and that both the total score and the scores at its four subscales are worthy of consideration. The physical–digital integration scores were found to be differently associated with digital and non-digital behaviors, individuals’ ability to read emotions in the facial expressions of others, and indicators of psychosocial functioning (anxiety, depression, and satisfaction with social relationships). The paper proposes a new measure whose scores are associated with several variables that may have relevant consequences at both individual and social levels.

## Introduction

In the last decade, web-based devices and technological applications have become ubiquitous and indispensable to carry out even the simplest daily activities, such as communicating, socializing, working, buying objects, getting information, and enjoying leisure time (Berman and Kesterson-Townes 2011; Line et al. 2011). In a few years, the capabilities and functions of these instruments have significantly advanced, particularly in terms of mobility, availability (i.e., 24/7), and capability to provide highly immersive experiences. Users can access the services offered by web-based devices at any time and from anywhere, and this has contributed to enormously increase their usage and perceived utility. The Internet of things, virtual reality, platforms for gaming, solutions for collaborative-working, and social network sites (SNSs) are only a few examples of what technology represents for people nowadays. All these instruments and applications contribute to transforming our living spaces (e.g., home, public places, offices, schools) into digitally enriched environments and making technological devices actual extensions of our bodies. The neologism “phygital” has been recently coined to indicate the new concept of space that originates from the growing convergence between the physical and digital dimensions. In this hybrid system, objects, tools, and bodies create a new scenario in which physical and digital data are highly mixed and, sometimes, not completely distinguishable (Gaggioli 2017). Digital, mobile and wearable devices, along with artificial intelligence (AI) applications, create a dynamic relationship between physical and digital realms. They allow for embedding digital objects and entities in the scene that people subjectively experience, so that individuals can no longer perceive the disconnection between physical and digital aspects of reality (De Souza and Silva 2006). The new environments that originate from the integration of digital and physical reality are globally referred to as extend reality. It includes virtual reality, augmented reality, and mixed reality, which are very common nowadays. Virtual reality defines fully computerized 3D environments in which users can interact with the scene and objects in a seemingly physical way. Augmented reality refers to environments that more strongly integrate the physical and digital realms. These environments are not characterized by an entirely digital scene, but they extend and complement physical reality with digital objects and cues. Mixed reality is a combination of physical and virtual realms that interact to create a new environment (Ziker et al. 2021).

Reality is nowadays increasingly represented by a mix of digital and physical cues, and people often experience the world not only through their body but also through the mediation of technological devices that have become essential instruments to interact with the environment (Dias 2014) and real “extensions of ourselves” (McLuhan et al. 2011). It could be supposed that the inclination to be immersed in this new hybrid system and not to perceive a real difference between physical and digital cues may depend on different factors, such as the frequency with which individuals use technologies, the attachment to the physical world, psychological or biological individual differences, or external pressures. Likewise, the outcome that may derive from the immersion into the hybrid reality may involve several aspects, such as subjective well-being or cognitive and social performances. Due to their relevant repercussions at social and individual levels, all these aspects deserve investigation and scientific attention.

This work aims to develop a scale for the assessment of a construct that we called physical–digital integration. This represents an individual difference that refers to the tendency of some individuals not to perceive a clear differentiation between data, perceptions, and feelings that pertains to physical and digital environments.

## Theoretical background

The present work moves from a detailed review of the literature that allowed for the identification of the main aspects that have been used to conceptualize the construct of physical–digital integration.

### Perceptions and interaction with the environment

According to recent findings in the literature, the immersion into a phygital world and the massive use of digital supports may influence our cognitions and our way to perceive and interact with the environment. For instance, several empirical studies showed that, compared with non-videogamers, people who highly use these supports perceive a greater amount of visual information (Green and Bavelier 2006), have better eye–hand coordination (Li et al. 2019) and higher processing speed (Greenfield 2009; Schmidt and Vandewater 2008). Other studies, on the other hand, stressed that the massive use of digital media, while improving perceptual skills, might weaken higher order functions, such as critical thinking, reflection, inductive problem solving, cognitive flexibility, inhibitory control and imagination (Aydın et al. 2020; Greenfield 2009). Another interesting finding pertains to the positive association between the use of digital devices and multitasking abilities (Cotten et al. 2014; Ettinger and Cohen 2020; Lenhart et al. 2015). Multitasking is taken to be one of the identifying characteristics of the “digital natives” who, having been born into a “phygital” world, have acquired the ability to direct their attention to several different sources of information, both digital and physical (Rideout 2015; Savina et al. 2017; Tapscott 2008). Research showed that 30% of young people multitask during their homework sessions, switching from academic tasks to digital media every 6 min (Foehr 2006; Rosen et al. 2013). Moreover, those who have more distracting technologies available have been found to be more inclined to multitask (Savina et al. 2017).

### Time and space perceptions

Scholars argued that the massive use of digital instruments may also influence the concepts of time and space. These two dimensions represent intrinsic and indispensable elements of physical reality. However, the penetration of technology in our lives leads to a reconceptualization of them (Kweon et al. 2011). Time and space have weakened. Indeed, nowadays human activities tend to take place through a series of acts that are realized in different realms across different times and spaces (Couclelis 2000, 2004; Schwanen and Kwan 2008). The virtual world, in fact, has given rise to new “electronic spaces” that diminished the relevance of spatial distances and changed the ways of interacting with the environment. In the modern world, we can perceive digital environments (e.g., online platforms, networking sites) as familiar as physical ones (e.g., homes, public rooms, offices), and we have learned to interact with the hybrid spaces around us using both the body and digital tools, thus giving less importance to physical distances. In the “global village”, interacting realistically, quickly, and effectively with anyone, wherever they are, has become very easy and common. Interestingly, not only the perception of space but also that of time has been strongly influenced by the spread of connectivity. Digitalization changed the dynamics of everyday life, the way in which time is organized, and ultimately our perceptions of time (Lee and Liebenau 2000; Tsatsou 2009). The digital life allows us to experience a different temporal dimension (also called “internet time”) in which individuals are always interconnected, and can collaborate, socialize, work, or buy services and objects even when it is not possible in physical spaces (e.g., online stores are open 24/7, work e-mails are accessible at any time). Recent studies demonstrated that the use of smartphone and media platforms may have a role in time distortion (Lin et al. 2015; Montag et al. 2015). For instance, it has been found that internet-addicted teenagers tend to have impaired spatial awareness and poor ability to be conscious of themselves in time (Katasonova et al. 2014). Other studies showed that using the Internet and mobile phone relaxes temporal constraints and enhances spatial flexibility (Schwanen and Kwan 2008).

### Social and Interpersonal Relationships

Becoming more and more integrated into everyday life, web-based devices are gaining a central role also in the management of social relationships (Haythornthwaite and Wellman 2002; Wellman and Gulia 1999). In fact, people have been found to use the Internet and other technological devices to create new relationships and to maintain the existing ones. Indeed, digital relationships are incorporated into the offline social circles (Hampton and Wellman 2002; Mesch and Talmud 2006; Parks and Floyd 1996), and non-digital relationships and digital networks have a large degree of overlap (Ellison et al. 2007; Subrahmanyam et al. 2008). In particular, it has been found that, generally, digital social relationships supplement rather than replace non-digital ones (Boyd and Ellison 2007; Burke and Kraut 2014; Ellison et al. 2007; Haythornthwaite 2002; Mesch and Talmud 2006). In this scenario, social and interpersonal relationships start and grow up relying upon a mixture of interactions that take place interchangeably in virtual or physical environments. According to the literature, the new way of experiencing social and interpersonal relationships has been found to have some repercussions on subjective well-being and individual functioning. For instance, some studies showed that the large use of SNSs might induce social comparisons and envy (Verduyn et al. 2017), jealousy in romantic relationships (Elphinston and Noller 2011), and large levels of depression (Aydin et al. 2020; Seabrook et al. 2016; Tandoc et al. 2015), stress (Chen and Lee 2013), and anxiety (Seabrook et al. 2016; Zaffar et al. 2015). In addition, the extensive use of social media has been associated with emotional exhaustion (Sriwilai and Charoensukmongkol 2016) and a reduced ability to accurately recognizing emotions (Ünal-Aydın et al. 2020). However, other studies also showed positive effects, such as reduced feelings of loneliness, improved life satisfaction (Deters and Mehl 2013; Masciantonio et al. 2021; Seabrook et al. 2016), and civic and political engagement (Valenzuela et al. 2009). The use of social media has also been found to be related to subjective well-being and feelings of social connectedness and support (McKenna and Bargh 2004, 2000; Hu et al. 2017; Shaw and Gant 2004; Verduyn et al. 2017), which in turn reduce stress and physical illness (Nabi et al. 2013). Other studies documented that the social contacts deriving from online chat sessions are associated with a reduction of loneliness and depression feelings and with improvements in perceived social support and self-esteem (Shaw and Gant 2004). It has also been found that, for socially anxious adolescents, Internet can be a valuable instrument to have intimate communications (Valkenburg and Peter 2007). Moreover, when social distancing measures were in place during COVID-19 pandemic, Internet use resulted in lower depression symptoms and higher quality of life for many people (Wallinheimo and Evans 2021).

### Self-identity

The enlargement of social relationships into the virtual arena is also associated with the topics of self-concept and identity development. In the first era of internet research, many scholars focused on the role of disembodiment and anonymity as aspects that may influence self-presentation and identity development (Bargh et al. 2002; McKenna et al. 2002). For instance, anonymity and the limited non-verbal cues that are typical of online interactions have been found to reduce social inhibition and anxiety, and to increase the propensity toward self-disclosure or the development of alternative-selves (Bargh et al. 2002; Derlega et al. 1993; Stern 1999). However, in recent years, the shift of SNSs to lower anonymity (e.g., some SNSs require an institutional e-mail thus not allowing for anonymity) posed new interest on the topic of self-presentation in the virtual scene. This is also due to the fact that SNSs are often used to interact with people belonging to the offline social network and who are, therefore, relevant in the negotiation of identity. The lack of anonymity of the new digital environments and the anticipation of subsequent face-to-face encounters have been hypothesized to narrow the discrepancy between ‘‘actual selves” and ‘‘alternative selves” in people’s online self-presentation (Ellison et al. 2006). Research seems to indicate that, despite some ‘‘truth-stretching” activities (e.g., posting photoshopped selfies, omitting some information), the identities developed on Internet are quite ‘‘realistic”, as users want to avoid unpleasant surprises in subsequent offline meetings (Ellison et al. 2006). Moreover, research suggests that users regard their online presentations as an integral part of their overall identity and, thus, seek to coordinate their online presentations with their offline self (Chen 2016; Zhao et al. 2008).

## The physical–digital integration scale (P-DIS) and the hypotheses concerning its nomological network

As highlighted by recent literature, the immersion into a world that is increasingly characterized by both physical and digital cues may influence our cognition and the way we perceive and interact with the environment. Drawing upon this evidence, the present work aims to develop a scale for the assessment of a construct that we called physical–digital integration. This represents an individual difference referring to the tendency of some individuals not to perceive a clear differentiation between data, perceptions, and feelings that pertain to physical and digital environments. The construct consists of four main facets: (a) the tendency to live social relationships through the use of both physical and digital interactions, without the need for the physical presence of interlocutors (social relationships); (b) The inclination to consider digital profiles and personal accounts as concrete manifestations of the self (identity); (c) The tendency not to perceive a clear differentiation between physical or digital aspects of the environment, thus considering digital devices as extensions of one’s senses (sensory perception); and (d) The tendency to have a distorted perception of time and space that, being embedded in the digital world, sometimes become excessively expanded or compressed (time–space perception).

Twenty-one items were developed to compose the *physical–digital integration scale* (P-DIS) and several analyses were performed to test its psychometric properties. In addition, different variables were considered to explore the nomological network of the construct, namely: the number of digital devices used daily, the time spent per day in non-digital recreational activities, indicators of psychosocial functioning (i.e., anxiety, depression, satisfaction with social relationships), and the ability to read emotions in the facial expressions of others.

Concerning the nomological network of the P-DIS, the following hypotheses were developed:

*H1: Higher scores at the P-DIS are associated with a larger use of digital devices and a shorter time spent in non-digital recreational activities.* This hypothesis is grounded in the idea that people who are more involved in digital environments and lower exposed to non-digital activities should develop a greater tendency to integrate physical and digital cues.

*H2: Higher scores at the P-DIS are associated with lower scores on the measures of psychosocial well-being.* The literature reported mixed results concerning the effect of using digital devices and SNSs in influencing psychological and social outcomes (e.g., Deters and Mehl 2013; Hu et al. 2017; Seabrook et al. 2016; Tandoc et al. 2015; Zaffar et al. 2015). However, we hypothesize that the tendency not to perceive a clear differentiation between physical and digital experiences may generate unpleasant feelings and confusion that may result in a lowering of psychosocial well-being.

*H3: Higher scores at the P-DIS are associated with a lower ability to properly recognize facial expressions of emotions*. People who highly integrate physical and digital cues should be inclined to pay attention to different information sources, that are not necessarily typical of the physical world. Thus, we hypothesize that, during social interaction, these individuals may encounter some difficulties to stay focused on and being effective in interpreting the facial expressions of others. These expectations are consistent with results of recent work that showed a negative association between SNS addiction and the ability to read emotions in the eyes of others (Ünal-Aydın et al. 2020).

### Generation and Selection of Items

An initial pool of 21 items was developed by the authors of the present work. Six items were developed for the P-DIS dimension investigating social relationships, whereas five items were developed for each of the remaining dimensions (i.e., identity, sensory perception, and time–space perceptions). The authors discussed the 21 items with scholars experienced in psychological assessment and social psychology. The discussion was aimed at evaluating if each item was clear and applicable to the intended dimension. Attention was also paid to develop subscales of equal length. Based on the discussion, 12 items were retained.

## Method: the psychometric properties of the P-DIS and its nomological network

### Participants

A total of 369 individuals (Males = 108, Females = 261; *M*_age_ = 32.32, *SD* = 12.15) from different Italian regions were enrolled through convenience sampling between February 2019 and April 2020. Participants were invited to fill out an electronic survey that was disseminated via e-mail (following a snowball procedure) and SNSs. Participants did not receive compensation for their participation in the study and the only inclusion criterion was being at least 18 years. Before accessing the online survey, participants were asked to agree with an electronic informed consent explaining the aim of the study, the duration of the task, and the possibility of withholding the consent to participate in the research at any time. Participation in the study was anonymous and voluntary. The study was carried out in compliance with the ethical principles for research established in the Declaration of Helsinki.

### Measures and procedure

After having answered a few sociodemographic questions, all participants were presented with the research questionnaire. It included four main sections, the first including the P-DIS, the second including three psychosocial measures (i.e., anxiety, depression, and satisfaction with social relationships), the third concerning the use of digital devices, and the last pertaining to facial emotion recognition.

P-DIS includes 12 items, grouped into four subscales (i.e., identity, social relationships, time–space perception, and sensory perception) containing three items each. All items were scored on a five-point Likert scale (from 1 “Completely disagree” to 5 “Completely agree”). The larger the score at the P-DIS and its subscales, the larger the level of integration.

Eight items, developed by the authors, were used to evaluate anxiety (e.g., “I feel tense and nervous”). Participants were asked to evaluate their habitual tendency to experience anxious feelings on a four-point scale (from 1 “Not at all” to 4 “Very much”). In this scale, the larger the score, the larger the level of anxiety. In the current sample, Cronbach’s *α* = 0.81.

The Italian version of the Patient Health Questionnaire-9 (PHQ-9; Kroenke et al. 2001; Spitzer et al. 1999) was administered to evaluate depressive symptoms according to the nine DSM-IV criteria. The questionnaire comprises nine items (e.g., “Feeling tired or having little energy”) scored on a four-point frequency scale (from 0 “Not at all” to 3 “Nearly every day”) that required respondents to evaluate how often they experienced depressive symptoms over the last 2 weeks. In this questionnaire, higher scores indicate higher levels of depression. This instrument has been found to have satisfactory reliability, sensitivity, and specificity (Kroenke et al. 2010). In the current sample, Cronbach’s *α* = 0.80.

Three items were administered to evaluate satisfaction with social relationships [e.g., “I feel satisfied with my social relationships”; see Colledani et al. (2021, 2022)]. The items were scored on a five-point Likert scale (from 1 “Strongly disagree” to 5 “Strongly agree”). In this scale, higher scores indicate higher satisfaction with social relationships. In the current sample, Cronbach’s *α* = 0.81.

Two items were used to evaluate the amount of time spent in non-digital recreational activities [“Please, indicate how many hours you generally spend in a day in non-digital recreational activities (e.g., happy hour with friends)”] and the number of digital devices used daily [“Please, indicate how many digital devices do you use daily (e.g., pc, tablet, smartphone, game console, home automation supports)”]. The first item was scored on a six-point scale from 1 “Less than one hour” to 6 “More than twelve hours”, the second item was scored on a six-point scale from 1 “Zero” to 6 “More than twelve”.

Finally, participants were presented with three pictures drawn from the face test developed by Baron-Cohen et al. (1997). The three pictures depicted the face of an actress who expressed three different emotions (i.e., happy, sad, and admiring). They were selected to represent one positive, one negative, and one neutral emotion. For all pictures, participants were asked to indicate the emotion that best represented the feeling expressed by the woman, choosing between two alternatives (i.e., disgust *vs* sad, surprise vs happy, and admiring *vs* surprise, for the pictures, where the actress was sad, happy, and admiring, respectively).

### Analyses

The factor structure of the P-DIS was investigated through factor analysis. Three models were tested and compared: a one-factor model, a correlated four-factor model, and a bifactor model. In the first model, all the 12 items of the scale loaded on a single dimension. In the second model, four different factors were defined (i.e., identity, social relationships, time–space perception, and sensory perception), each consisting of three items. The four factors were allowed to correlate. Finally, a bifactor model was run that included one general factor (i.e., physical–digital integration) measured by all the 12 items of the scale, and four domain-specific factors (i.e., identity, social relationships, time–space perception, and sensory perception), each measured by three items. All models were run using Mplus7 (Muthén and Muthén 2012), and the maximum likelihood estimator with adjusted means and variances [MLM; Muthén and Muthén (2012); see also Asparouhov and Muthén (2010), Hoyle (2012) and Satorra and Bentler (1994)], that provides standard errors and statistical tests that are robust to non-normality.^1^

The goodness-of-fit of the three models was evaluated using several fit indices: *χ*^2^, Comparative Fit Index (CFI; Bentler 1990), Standardized Root Mean Square Residual (SRMR; Bentler 1995), Root Mean Square Error of Approximation (RMSEA; Browne and Cudeck 1993), and Akaike Information Criterion (AIC; Akaike 1974). A non-significant *χ*^2^ (*p* ≥ 0.05) suggests adequate fit. However, it is well-known that *χ*^2^ is sensitive to sample size. Therefore, the other fit measures were considered in evaluating the models. Specifically, CFI above 0.90 (over 0.95 for excellent fit), SRMR less than 0.08, and RMSEA smaller than 0.08 (0.06 to 0.08 for reasonable fit) are indicative of satisfactory goodness-of-fit (Brown 2006; Hu and Bentler 1999; Marsh et al. 2004). Concerning AIC, smaller values are indicative of a better fit. Relative differences were considered meaningful if models differed in AIC by 10 or more (Burnham et al. 2011).

The Explained Common Variance (ECV; Sijtsma 2009; Ten Berge and Sočan 2004) is the ratio of the common variance explained by the general factor to the total common variance (Reise et al. 2013a, b; Rodriguez et al. 2016). High values (0.70–0.80) indicate that the factor loadings obtained from a unidimensional model might approximate well (i.e., be unbiased) the factor loadings on the general factor obtained from a bifactor solution, and suggest that the scale is substantially unidimensional (Rodriguez et al. 2016). For the general factor, McDonald’s (1999) omega (*ω*) and omega hierarchical (ωh) coefficients were also computed. These coefficients are factor-analytic “model-based” estimates of internal consistency. The former represents the proportion of variance in the scores that can be attributed to all sources of variance (i.e., general and domain-specific factors), whereas the latter quantifies the amount of variance that is accounted for by the general factor (Reise et al. 2010; Revelle and Zinbarg 2009; Zinbarg et al. 2005, 2007). Values of ωh larger than 0.75–0.80 indicate that a factor can be interpreted as the measure of a single construct despite multidimensionality (Reise et al. 2013a, b). McDonald’s (1999) ω and Cronbach’s α were computed for all scales. For both indices, values close to or greater than 0.70 denote satisfactory internal consistency (e.g., Kline 1998; Nunnally 1978).

The nomological network of the P-DIS was evaluated by inspecting the correlations of the total score of the instrument and those of its four subscales with other variables, such as: the number of digital devices used daily, the time spent in non-digital recreational activities, the levels of depression and anxiety, the levels of satisfaction with social relationships, and the ability in reading emotions in facial expressions. According with research hypotheses, negative associations were expected between the P-DIS scores and the time spent on non-digital recreational activities. Conversely, positive relationships were expected with the number of digital devices used daily. The P-DIS was also expected to have positive associations with depression and anxiety, and negative associations with the ability to read emotions in facial expressions and with satisfaction with social relationships.

## Results

The descriptive statistics of all the used variables are reported in Table 1, whereas the results of the factor analyses are shown in Table 2. The one-factor model obtained an acceptable fit (*χ*^2^(54) = 198.573, *p* < 0.001; RMSEA = 0.09 [0.07, 0.10]; CFI = 0.90; SRMR = 0.06; AIC = 14,182.455) with all items exhibiting high loadings on the factor (*λ*s from 0.39 to 0.76, *p*s ≤ 0.001). However, the other two models obtained a better fit (four-factor model: *χ*^2^(36) = 140.038, *p* < 0.001; RMSEA = 0.07 [0.06, 0.09]; CFI = 0.93; SRMR = 0.05; AIC = 14,127.187; bifactor model: *χ*^2^(36) = 140.038, *p* < 0.001; RMSEA = 0.07 [0.06, 0.09]; CFI = 0.93; SRMR = 0.05; AIC = 14,127.187). In the four-factor model, consistently with theoretical expectations, all items showed meaningful loadings on the intended dimensions (*λ*s from 0.40 to 0.83, *p*s ≤ 0.001) and correlations between factors were large (*r*s = from 0.70 to 0.94, *p*s ≤ 0.001). With regard to the bifactor model, all items significantly loaded on the general factor (*λ*s = from 0.36 to 0.90, *p*s ≤ 0.001) and on the relative domain-specific factors (*λ*s from 0.15 to 0.64, *p*s ≤ 0.05). Only for the subscale identity, one item (i.e., Item 2) did not significantly load on the relative domain-specific factor, even if it loaded on the general factor.

**Table 1:** Descriptive statistics

Scale	*N*	*Mean*	*SD*
P-DIS–total score	369	3.03	0.90
P-DIS–identity	369	3.11	1.18
P-DIS–social relationships	369	3.01	1.11
P-DIS–time–space perception	369	3.07	1.04
P-DIS–sensory perception	369	2.95	1.04
Satisfaction with social relationships	369	2.57	1.16
PHQ-9	369	0.80	0.47
Anxiety	369	2.60	0.61
Number of digital devices used daily	369	2.97	1.35
Time spent daily on non-digital recreational activities	369	2.32	1.12
Face test	261	2.89	0.34

*P-DIS* physical–digital integration scale, *PHQ-9* patient health questionnaire-9

**Table 2:** Results of factor analyses

	Unidimensional model	Bi-factor model					Correlated four-factor model			
		General	Identity	Social relationships	Sensory perception	Time–space perception	Identity	Social relationships	Sensory perception	Time–space perception
(1) Transforming the look of my internet profiles is like refreshing my appearances, like a new haircut or a new dress	0.739	0.599	0.643				0.753			
(2) Some part of me is on my devices	0.760	0.901	− 0.034^†^				0.769			
(3) My social image is actually a part of me	0.711	0.647	0.36				0.738			
(4) I like sharing the significant events of my life on social media, because it makes me feel as if everyone who matters to me could participate in them	0.631	0.511		0.437				0.665		
(5) When I have troubles or I am tense, speaking with my loved ones on social networks comforts me as if they were with me	0.599	0.474		0.431				0.634		
(6) I have friendships that I could define digital, because we keep continuously in touch but mainly through social media	0.583	0.510		0.306				0.605		
(7) Nowadays, digital devices are real extensions of our body	0.385	0.399			0.244**				0.458	
(8) I would say that physical and digital data are fused in an indissoluble mix	0.710	0.704			0.321				0.834	
(9) Nowadays, there is no longer a clear distinction between physical and digital data	0.450	0.363			0.533				0.511	
(10) Time and space become less rigid when we are connected	0.417	0.385				0.146*				0.404
(11) There are places on the web that are as familiar to me as my home	0.608	0.472				0.586				0.724
(12) The possibility of partly living on the web extends the time of a day by a lot	0.595	0.463				0.563				0.718
Correlation between factors										
Sensory perceptions						0.375**				0.703
Social relationships					0.231^†^	0.684			0.733	0.835
Identity				0.726	0.231^†^	0.375**		0.938	0.828	0.766

All parameters were significant at *p* ≤ 0.001, excluding those indicated with *p ≤ 0.05, **p ≤ 0.01, ^†^*p* > 0.05

The inspection of differences in AICs (ΔAIC) indicates that the bifactor model was superior compared with the other two models (ΔAIC between the one-factor and correlated four-factor models = 55.27; ΔAIC between the one-factor and bifactor models = 94.81, and ΔAIC between the correlated four-factor and bifactor models = 39.54). Moreover, given the high correlations between the latent factors in the correlated four-factor model (*r*s from 0.70 to 0.94, *p*s ≤ 0.001), the bifactor solution seems to be the most suitable option to represent the structure of the scale. Indeed, in the bifactor model, the four domain-specific factors represent different facets of a general construct that is captured by the general factor.

The ECV of the bifactor model provided additional support to the multidimensionality of the scale. This index reflects the amount of bias in the parameter estimates that can be observed when multidimensional constructs are forced into a unidimensional model. When ECV values are greater than 0.70, a scale can be considered as essentially unidimensional (Rodriguez et al. 2016). The ECV of the P-DIS was 0.64, indicating that the scale should be intended as multidimensional. However, the value of the ωh coefficient for the general factor was high (0.79). This result suggests that, despite multidimensionality, the general factor is strong enough to be interpreted as the measure of a single construct (Reise et al. 2013a, b).

With regard to internal consistency, ω and α coefficients were satisfactory for the P-DIS–total score (*ω* = 0.88, *α* = 0.87) and acceptable for the subscales (ωs = 0.66, 0.87, 0.67, 0.66; αs = 0.63, 0.80, 0.67, 0.63 for identity, social relationships, sensory perception, and time–space perception).

On the whole, the correlations between the P-DIS scores and the other variables were in line with the expectations (see Table 3). The P-DIS–total score and the scores at subscales identity and relationships were positively associated with the number of digital devices used daily, while all P-DIS scales were negatively associated with the time spent daily on non-digital recreational activities. Positive correlations were found between all P-DIS scales and anxiety, whereas a weak negative association was observed between the subscale relationships and depression. Finally, negative associations were found between all P-DIS scales and satisfaction with social relationships and, except for the subscale time–space perception, also with the ability to recognize emotions in facial expressions. With regard to emotion recognition, the negative associations mainly pertained to the recognition of sad (negative emotion) and admiring (neutral emotion) expressions.

**Table 3:** Correlations between P-DIS scales and other measures

	P-DIS–total score	Identity	Social relationships	Time–space perception	Sensory perception
PHQ-9	− 0.069	− 0.052	− 0.107^*^	0.019	− 0.083
Anxiety	0.501^***^	0.412^***^	0.449^***^	0.440^***^	0.337^***^
Time spent daily on non-digital recreational activities	− 0.187^***^	− 0.163**	− 0.128*	− 0.142^**^	− 0.181^***^
Number of digital devices used daily	0.135^**^	0.116^*^	0.138^**^	0.090	0.098
Satisfaction with social relationships	− 0.332^***^	− 0.252^***^	− 0.290^***^	− 0.334^***^	− 0.214^***^
Face test—total score	− 0.183^**^	− 0.146^*^	− 0.163^**^	− 0.107	− 0.148^*^
Face test—sad face	− 0.159^*^	− 0.143^*^	− 0.128^*^	− 0.103	− 0.116
Face test—happy face	0.021	0.030	− 0.012	0.019	0.026
Face test—admiring face	− 0.134^*^	− 0.093	− 0.121	− 0.068	− 0.132^*^

*P-DIS* physical–digital integration scale, *PHQ-9* patient health questionnaire-9

*p < 0.05, **p < 0.01, ***p < 0.001

## Discussion

In the present work, a scale for the assessment of a construct that has been called physical–digital integration was developed. The construct refers to the tendency of some individuals not to perceive a clear differentiation between feelings and perceptions that pertain to physical and digital environments. The construct has been articulated in four facets, namely, identity, social relationships, time–space perception, and sensory perception.

The scale includes 12 items. The analyses performed to evaluate the psychometric characteristics of the scale showed that it is well-represented by a bifactor structure, with a general factor and four domain-specific factors corresponding to the four aforementioned facets. The indices associated with the bifactor model supported the non-unidimensionality of the scale and, at the same time, showed that the general factor, despite multidimensionality, has sufficient strength to be considered a reliable measure of the general construct. Thus, both the use of the total scale score and that of the domain-specific scores were supported. Internal consistency indices (*α* and *ω*) were acceptable for all scales, and correlational analyses supported nomological validity. In particular, according to the hypotheses, negative associations were found between all physical–digital integration scales and the time spent daily on non-digital recreational activities. Conversely, the number of digital devices used daily was positively associated with the P-DIS–total score and the scores at the subscales identity and relationships. These results supported the validity of the scale: the more people use digital instruments and the less they engage in non-digital activities, the more they report difficulties in distinguishing physical and digital experiences.

Interesting findings also derive from the inspection of the correlations between the P-DIS scores and the other considered measures. In particular, according to our expectations, all P-DIS scores were positively associated with anxiety and negatively associated with satisfaction with social relationships. Conversely, concerning depression, only a weak yet significant correlation was observed with the physical–digital integration subscale that pertains to social relationships. In contrast to our expectations, the direction of this correlation was negative, thus indicating that, the more people have difficulties in distinguishing between physical and digital relationships, the less depressed they feel. This result is in contrast with our hypotheses. It should be noted that, in the literature, there are mixed results on the relationship between the use of digital technologies and depression, with some studies finding positive associations (e.g., Cotten et al. 2012; Seabrook et al. 2016; Tandoc et al. 2015) and others negative associations (e.g., Cotten et al. 2012). Moreover, it should be noted that, in our sample, PHQ-9 scores were very low, this indicating that our participants were in general not depressed. Future research should be devoted to investigating if a different result is observed in samples with higher levels of depression. Future research should also explore potential positive consequences of digital integration of social relationships on individuals’ well-being.

Interestingly, the P-DIS–total score and the scores at the subscales showed different patterns of associations with the other considered measures. This provides support for the usefulness of considering both the score on the general factor and those on the domain-specific factors.

Another highly interesting result regards the negative association that was found between the scores on the P-DIS (i.e., the P-DIS–total score and the scores at the social relationships, identity, and sensory perception domain-specific scales) and the score on the face test. These correlations suggest that those individuals who do not perceive a clear differentiation between physical and digital experiences might be less able to interpret the emotions of their interlocutors. A similar result was also observed in a recent study by Ünal-Aydın et al. (2020), who found an association between social network addiction and poor abilities in identifying emotions from eye expressions. According to our hypotheses, the impaired ability of people who highly integrate physical and digital cues in recognizing facial emotions may be due to their ways of processing information. In particular, we hypothesized that, when these individuals evaluate the emotions of their interlocutors, they are more inclined than others to pay attention to several sources of information, which are not necessarily anchored on the physical world. Thus, people who highly integrate physical and digital data may be less effective in recognizing the adequate cues in the faces of others. This explanation is reasonable also taking into account the findings of a study by Ge et al. (2017), that showed how internet-addicted individuals adopt a facial emotion information processing mode that differs from that adopted by non-addicted people (e.g., longer fixation durations, lower fixation counts, and uniform extraction of pictorial information). These findings are worthy of further research attention, since the ability in recognizing emotions may have relevant consequences at both individual and social levels. At the individual level, the difficulty in recognizing emotions in the face of others could generate problems in coordinating with them and in creating close and satisfying relationships. At the social level, this could lead to an impoverishment of culture, social relationships, and interpersonal and intergroup solidarity. The massive use of video conferencing, which in recent years has become necessary due to the COVID-19 pandemic, could have further influenced our way of interpreting emotional cues. In this scenario, the challenge for the next years is to develop solutions, applications, and devices that facilitate the emotional exchange between the interlocutors. AI could be the most valid tool for this purpose. A limitation of the study is that only self-report scales were used and that two of them (those used to evaluate satisfaction with social relationships and anxiety) did not undergo full validation. Moreover, only some items of the face test were used. Finally, since participants were recruited only through online procedures, they may not be representative of the population.

Another limitation of the present work relies on its cross-sectional nature. The physical–digital integration facets were found to be associated with depression, anxiety, and satisfaction with social relationships. However, these variables could be either antecedents or outcomes of physical–digital integration. Future studies with a longitudinal design may help to clarify causal relationships between these variables. Future studies should investigate the invariance of the P-DIS across gender, age classes, or culture of respondents (Colledani 2018; Colledani et al. 2018a, b, 2019b), and might investigate possible latent profiles underlying the physical–digital integration facets. The latter analysis may help to obtain a holistic interpretation of physical–digital integration, and may be useful in applied settings to clearly understand and describe individual differences in this construct (Colledani et al. 2019a, b; Dal Corso et al. 2020; Ferguson and Hull 2018).

## Conclusion and final remarks

In the last decades, the penetration of technology in our daily lives has progressively increased, giving raise to environments that are a mix of digital and physical cues. Some individuals still tend to perceive a clear differentiation between the two realms, while others tend to experience a greater level of fusion between them. This can have both positive and negative effects on psychological and social well-being. Moreover, the tendency to highly integrate physical and digital cues can impact the ability to correctly recognize facial emotion expressions. These effects are mainly present in those individuals who often use technology in their daily lives.

As a consequence of the COVID-19 pandemic, the health emergency fueled the adoption of digital solutions as it has never happened before, creating new opportunities to expand digital approaches to social, economic, and cultural life (Hantrais et al. 2021; Motiejūnaitė-Schulmeister and Crosier 2020). In this condition, it could be expected that the tendency of some individuals to integrate digital and physical realms has strengthened. Future research should explore this, as well as its potential consequences on psychological and social well-being and attitudes. Research should also investigate the readiness of different social groups to adapt to the increased penetration of technology in daily life. Indeed, socio-economic, gender, age, and ethnic differences could have influenced the possibilities and inclinations of some individuals to access and use digital devices and applications as it is required nowadays (Aissaoui 2021; Allmann 2020; Motiejūnaitė-Schulmeister and Crosier 2020; Holmes and Burgess 2020). For example, young people have had many opportunities to use new solutions for productive and social life and, consequently, they are expected to be able to move fluently between the digital and physical realms. In contrast, due to attitudes, personal motivations, education, and income, the elderly tend to be more excluded from the digital life (Song, et al. 2021). While this problem is not new, it became more relevant during the COVID-19 pandemic, because many individuals were unable to use those digital measures that were put in place to help them (Van Jaarsveld 2020).

With this in mind, future research should be addressed to make technology more usable by those classes of individuals who are currently excluded from its fruition. Moreover, it is crucial to investigate the impact that the increasing fusion of digital and physical realms may have on subjective well-being and social performances. AI applications could be extremely useful to face both these aspects. AI consists of several technologies, such as machine learning, robots, avatars, and touchscreen intelligent devices (Balakrishnan and Dwivedi 2021; Grewal et al. 2020; Huang and Rust 2018; Pillai et al. 2020; Vimalkumar et al. 2021) that could be used to improve the integration between physical and digital realms, also facilitating the inclusion in the digital environments of those people who are currently on the fringes (Hantrais et al. 2021; Vimalkumar et al. 2021). AI should also be used to develop new solutions aimed to facilitate the emotional exchange among technology users.

Human beings are helped in their social interactions by emotions, which represent a universal language able to overcome cultural diversities. Indeed, facial expressions are responsible for transmitting valuable information, which was difficult to communicate otherwise. Emotions express the mental state of individuals, and this is directly related to their intentions, conditions, and feelings. Missing emotional information represents a relevant loss in communication.

## Data Availability

The data used to support the findings of this study are available upon request from the corresponding author.

## Footnotes

1 An a-priori analysis indicated a minimum sample size of *N* = 308 to obtain an adequate power in a model with 5 latent factors and 12 observed indicators (Type-I error *α* < 0.05, effect size = 0.30, power = 0.80; see Soper 2020).

## Materials availability

The materials used to support the findings of this study are available upon request from the corresponding author.

## Code availability

The codes used to support the findings of this study are available upon request from the corresponding author.
